# Analysis of Melt Pool Characteristics and Process Parameters Using a Coaxial Monitoring System during Directed Energy Deposition in Additive Manufacturing

**DOI:** 10.3390/ma12020308

**Published:** 2019-01-19

**Authors:** Christian Kledwig, Holger Perfahl, Martin Reisacher, Frank Brückner, Jens Bliedtner, Christoph Leyens

**Affiliations:** 1 Development Department, Sauer GmbH LASERTEC, DMG MORI AG, 87459 Pfronten, Germany; holger.perfahl@dmgmori.com (H.P.); martin.reisacher@dmgmori.com (M.R.); 2 Product and production development, Luleå University of Technology, 971 87 Luleå, Sweden; Frank.Brueckner@iws.fraunhofer.de; 3 Additive Manufacturing and Printing, Fraunhofer Institute for Material and Beam Technology IWS, 01277 Dresden, Germany; Christoph.Leyens@iws.fraunhofer.de; 4 SciTec Department, Ernst-Abbe-Hochschule Jena, 07745 Jena, Germany; Jens.Bliedtner@eah-jena.de; 5 Institute of Materials Science, Technische Universität Dresden, 01062 Dresden, Germany;

**Keywords:** directed energy deposition, laser metal deposition, laser cladding, process parameters, melt pool, coaxial monitoring, process monitoring

## Abstract

The growing number of commercially available machines for laser deposition welding show the growing acceptance and importance of this technology for industrial applications. Their increasing usage in research and production requires process stability and user-friendly handling. A commercially available DMG MORI LT 65 3D hybrid machine used in combination with a CCD-based coaxial temperature measurement system was utilized in this work to investigate what information relating to the intensity distribution of melt pool surfaces could be appropriate to draw conclusions about process conditions. In this study it is shown how the minimal required specific energy for a stable process can be determined, and it is indicated that the evolution of a plasma plume depends on thermal energy within the base material. An estimated melt pool area—calculated by the number of pixels (NOP) with intensities larger than a fixed, predefined threshold—builds the main measure in analysing images from the process camera. The melt pool area and its temporal variance can also serve as an indicator for an increased working distance.

## 1. Introduction

Directed energy deposition (DED) is based on a laser process in which a laser beam generates a melt pool on a substrate. An additional metallic powder material is transported to the melt pool, where it becomes molten. Due to a feed movement, the molten material cools down and welding tracks are formed. By placing a couple of single tracks side by side, a planar coating can be obtained. Furthermore, it is possible to generate whole three-dimensional structures by depositing one layer or track at the top of another already welded layer or track [1].

This technique can be used to coat or repair already existing parts, as well as to create completely new components. Many advantages, like a much higher deposition rate or no longer need of support structures compared to powder bed techniques, lead to a growing importance for industrial applications [2]. On the other hand, the variety of parameters leads to a high complexity and requires a deep understanding of the process [1].

A great deal of research has been done to analyse the effect on the cladding track according to a systematic variation of process parameters [3,4,5,6]. For example, de Oliveira et al. [4] showed that the clad width W increases as the energy per unit length (given as $P/v_{f}{}^{1/2}$, with the laser power P and scanning speed $v_{f}$) increases. On the other hand, the authors determined an increasing clad height H, with an increasing powder mass per length $\dot{m}/v_{f}$, with the powder feed rate $\dot{m}$ and scanning speed $v_{f}$.

In current practice, most suitable measurement systems work contactless and using optical technology [7]. Several studies [8,9,10,11] have shown that temperature distribution inside a melt pool, as well as its shape, contain information about process parameters. Meriaudeau et al. [9] determined a decreasing melt pool surface temperature as an indicator for an increasing powder feed rate $\dot{m}$. Walter [10] uses melt pool size A as an input for a closed-loop control to adjust the laser power P. Ocylok et al. [8] showed the influence of the laser power P, scanning speed $v_{f}$, powder feed rate $\dot{m}$ and temperature of the base plate θ on melt pool size A, where the laser power P had the biggest influence (a 346% larger melt pool size by doubling the laser power from P=700 W up to P=1400 W).

The previously cited references mostly focus on single tracks. There, the heat transfer situation can be assumed as constant, which is not the case when complex 3D geometries are built up. Furthermore, the aforementioned publications predominantly analyse experiments performed in specially developed test stations under laboratory conditions. 

This article examines the influence of the process parameters laser power P, scanning speed $v_{f}$, powder feed rate $\dot{m}$ and substrate plate–nozzle distance Δz on the melt intensity distribution of the melt pool surface while building up a single-track multilayer fillet.

## 2. Materials and Methods 

### 2.1. Laser Metal Deposition Experiments

**Figure 1:**
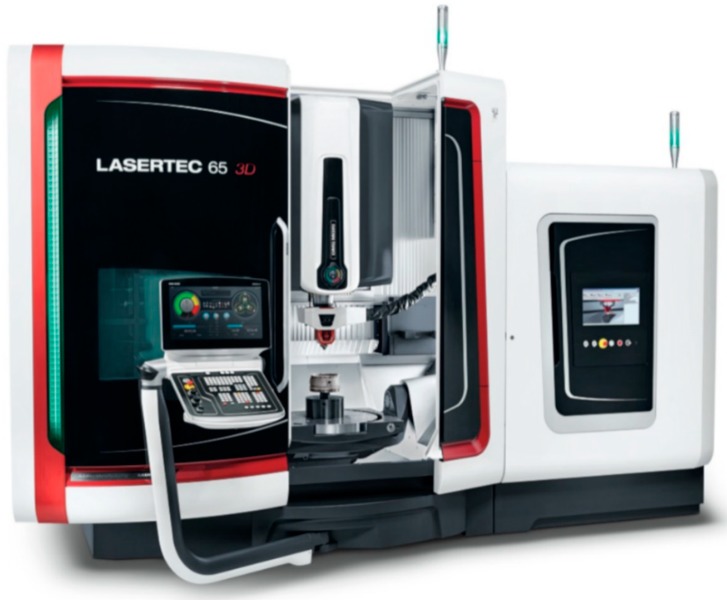
DMG MORI Lasertec 65 3D *hybrid* machine.

**Figure A1:**
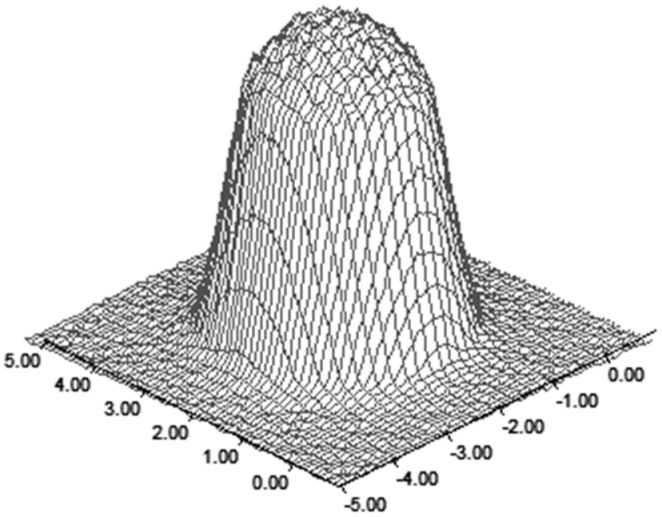
Measured laser intensity profile using a Primes FocusMonitor FM 120.

All laser deposition experiments were performed on a commercially available DMG MORI Lasertec 65 3D hybrid machine (LT 65 3D hybrid, DMG MORI AG, Pfronten, Germany), a five-axis hybrid machine that combines the additive DED process with subtractive milling and turning techniques (Figure 1). The machine includes a diode laser (wavelength λ=1020 nm) with a maximum power of P=2500 W and a Coax 9 (https://www.iws.fraunhofer.de/de/geschaeftsfelder/thermische_oberflaechentechnik/auftragschweissen/systemtechnik.html; accessed on 16.11.2017) powder nozzle. Furthermore, it was equipped with a disc powder feeder (11 mm×0.6 mm gouge). The spot diameter of the laser beam was d=3.0 mm at a focal length of f=200 mm, with a top hat intensity profile (Figure A1). As cladding material, gas-atomised stainless steel powder (X2CrNiMo17-12-2 by Carpenter Powder Products), which is mostly spherical in shape and has a particle size of 45−105 μm, was used. 

A hollow cylinder (diameter ∅=100 mm, height h=30 mm) was used as the experimental geometry, which was produced with a helical, single track build-up by rotating the C-axis and increasing the height (Z-axis) continuously. In this way it combined a changing heat transfer situation during the process without disturbance during starts and stops. A cylinder builds the simplest geometry that can be used in realistic applications. Furthermore, the stable and reproducible process conditions formed a solid basis for data acquisition. 

In this study, 24 hollow cylinders (as defined above) were deposited on mild steel plates (material: S235JR; dimensions: ∅100 mm×10 mm) by varying the four process parameters: laser power P, scanning speed $v_{f}$, powder feed rate $\dot{m}$ and substrate plate–nozzle distance Δz. The range of variation is listed in Table A1. During the alternation of one parameter, the other parameters remained constant at the standard values: P=1800 W, $v_{f}=1000\;\mathrm{mm}/\min$, $\dot{m}=14\;g/\min$ and Δz=11 mm.

### 2.2. Melt Pool Intensity Distribution Acquisition and Data Analysis

**Figure 2:**
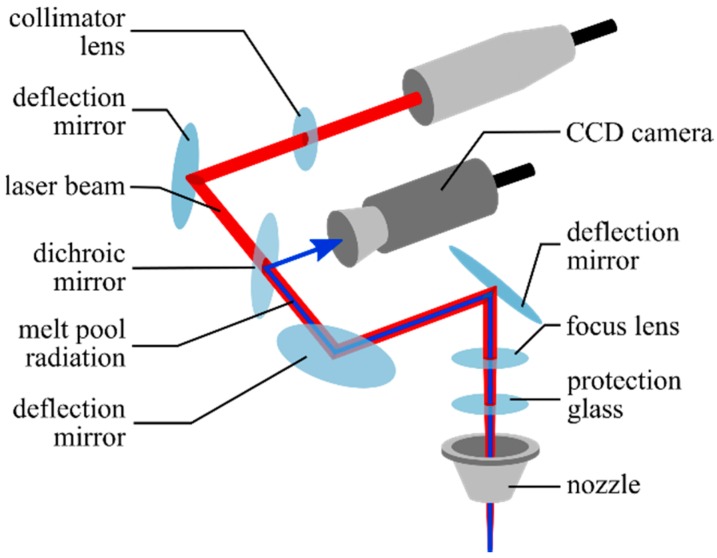
Optical path of laser beam and melt pool radiation inside the laser head of a Lasertec 65 3D *hybrid* machine.

**Figure 3:**
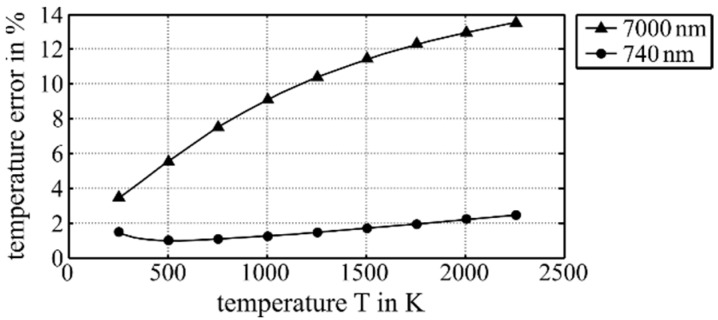
Relative error in temperature estimation using a 20% wrong emissivity value at the different wavelengths 740 nm (bandpass filter used during this research) and 7000 nm (usual wavelength for infrared thermal measurements). The figure is a result of Planck’s law and was calculated by numerical integration.

The intensity distribution of the melt pool was monitored by using a CCD camera-based temperature measuring system, which is an optional part of the machine. It observed radiation at a wavelength of 740 nm. The system was placed inside the laser head in which a dichroic mirror reflects the melt pool radiation to the camera (Figure 2). The wavelength spectrum used was suitable to analyse the melt pool temperature because of the nearly independent emissivity on this wavelength (Figure 3). The camera was connected to a PC via Ethernet, where the intensity distribution was documented as monochrome pictures at a sampling rate of about 2 Hz. 

**Figure A2:**
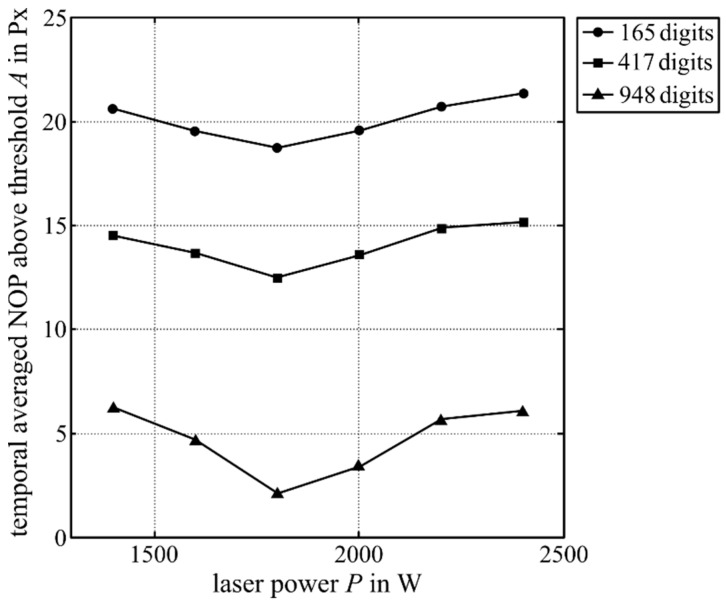
Temporally averaged NOP (over the intensity thresholds 163 digits, 417 digits and 948 digits) over the laser power variation. The three curves follow the same characteristic, which is most pronounced in the curve of 948 digits.

The camera system was calibrated by applying an LED-based calibration emitter. According to the individual calibration of the used machine, an intensity value of 163 digits represented the temperature of *T* = 1672 K, which is the liquidus temperature of X2CrNiMo17-12-2. To calculate the melt pool area (see Walter [10]) a threshold needed to be predefined, above which a pixel is counted to the melt pool area. Due to additional radiation from surface plasma [11,12], the threshold of 163 digits led to overexposed camera images. As an example, it is shown in Figure A2 that the characteristic of the temporally averaged number of pixels (NOP) over the laser power variation did not change with the threshold value. On the other hand, with a threshold of 948 digits the characteristic of the temporally averaged NOP could be seen in the most detailed way. Therefore, a threshold of 948 digits was chosen as the object criterion to compare the results during this article.

**Figure 4:**
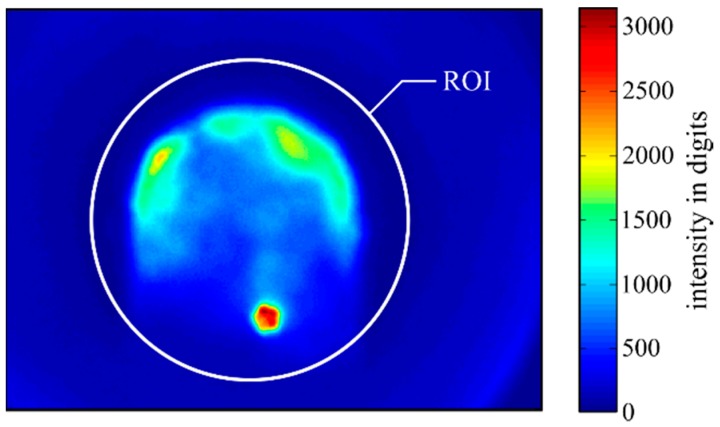
Example image in false colours, taken during the variation of laser power at P=1800 W. It shows the intensity distribution of the melt pool. To cut away the misdirected melt pool radiance (light blue), a region of interest (ROI) was adjusted and set to the position of the nozzle opening.

All data analysis was done with image processing. The first step was to apply a circular region of interest (ROI) to cut away misdirected melt pool radiance (Figure 4). According to [8], the number of pixels over a predefined threshold was chosen to characterise the images (in a camera image without artefacts from the process, like powder particles or plasma radiation, an intensity threshold of 163 digits would indicate the melt pool size).

## 3. Results

### 3.1. Steady State and Reproducibility

**Figure 5:**
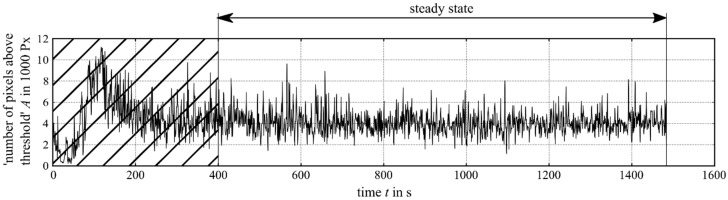
The number of pixels (NOP) within the ROI above a threshold of 948 digits calculated for a whole cylinder build-up. The first quarter of the signal was not considered for the calculations because of its transient behaviour caused by the heat-up dynamics. The graph shows the experiment at P=2000 W.

Because of the changing heat transfer situation, it is important not to consider all frames of an experiment, but to consider just the frames during steady state (Figure 5). In this case, we recognised a significant difference between the first-layer signal (t=0−50 s), the transient behaviour (t=50−400 s) and the steady-state signal (t=400−1500 s).

The first experiments were conducted to ensure reproducibility. Therefore, three cylinders were built up using the standard parameters (P=1800 W, $v_{f}=1000\;\mathrm{mm}/\min$, $\dot{m}=14\;g/\min$ and Δz=11 mm). The temporal average of the NOP showed a maximum deviation of 0.39%, while the temporal standard deviation of the NOP showed a deviation of 9.74%. Based on this result we concluded that one execution of each experiment was representative enough to derive meaningful results.

### 3.2. Variation of the Laser Power

Within the experiments, the laser power was varied from P=1400 W to P=2400 W in increments of 200 W. This range was chosen because the process became unstable (“unstable“ denotes a process that leads to dilution problems or porosity problems) at a power range of P<1400 W.

**Figure 6:**
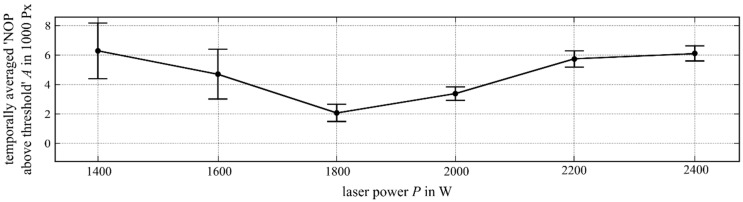
Temporally averaged NOP (over an intensity threshold of 948 digits) over the laser power variation. The temporal standard deviation is shown for each experiment.

The temporally averaged NOP showed a maximum value of 6286 Px at a power of P=1400 W (Figure 6). From that point, increasing laser power led to a local minimum (2153 Px) at P=1800 W before increasing again. The maximum of the temporal standard derivation (59.5%) appeared at P=1400 W. It could be observed in the experiments that the welding process showed a behaviour with increased stochasticity for laser powers P<1800 W. This was reflected by the higher standard deviation for P=1400 W and P=1600 W. Detailed analysis revealed that at P=1400 W and P=1600 W the distribution of observed NOPs was bimodal.

### 3.3. Variation of the Scanning Speed

Motivated by common industrial applications, the variation range of the scanning speed was chosen as $v_{f}=800-1600\;\mathrm{mm}/\min$.

**Figure 7:**
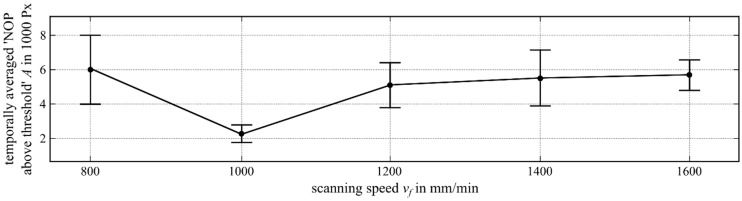
Temporally averaged NOP (intensity threshold 948 digits) over the scanning speed variation.

Considering the scanning speed variation (Figure 7), the maximum temporally averaged NOP (6032 Px) occurred at a scanning speed of $v_{f}=800\;\mathrm{mm}/\min$. By increasing the scanning speed, the temporally averaged NOP decreased, reaching its minimum at $v_{f}=1000\;\mathrm{mm}/\min$, while at higher scanning speeds it rose again to 5702 Px at a scanning speed of $v_{f}=1600\;\mathrm{mm}/\min$. Throughout the whole variation, a standard deviation between 33% and 66% could be seen, which became maximal at a scanning speed of $v_{f}=800\;\mathrm{mm}/\min$ (66%).

### 3.4. Variation of the Powder Feed Rate

As with the range of the scanning speed variation, the powder mass flow variation range was set to $\dot{m}=8-18\;g/\min$ base on common industrial applications.

**Figure 8:**
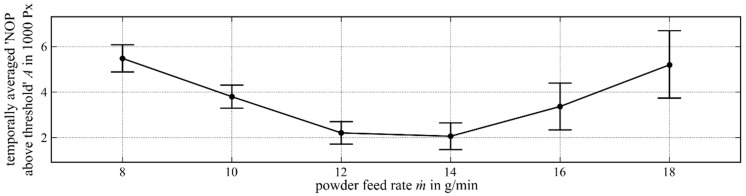
Temporally averaged NOP (intensity threshold 948 digits) over the powder feed rate variation.

The graph of the temporally averaged NOP over the powder feed rate variation (Figure 8) starts with 5489 Px at a powder feed rate of $\dot{m}=8\;g/\min$. With an increasing powder feed rate, the temporally averaged NOP decreased so that it reached its minimum of 2059 Px at a powder feed rate of $\dot{m}=14\;g/\min$. The temporally averaged NOP then increased up to a value of 5199 Px at a powder feed rate of $\dot{m}=18\;g/\min$.

The relative temporal standard deviation increased with the growing powder feed rates until $\dot{m}=12\;g/\min$. With a further increasing powder feed rate, it stayed almost constant at about 50%.

### 3.5. Variation of the Substrate Plate–Nozzle Distance

The range of the substrate plate–nozzle distance variation was chosen as Δz=8−20 mm. Distances of Δz<8 mm resulted in a growing nozzle temperature and thus to a growing number of nozzle adhesions, which disturbed the process. At distances of Δz>20 mm, no welding tracks could be formed.

**Figure 9:**
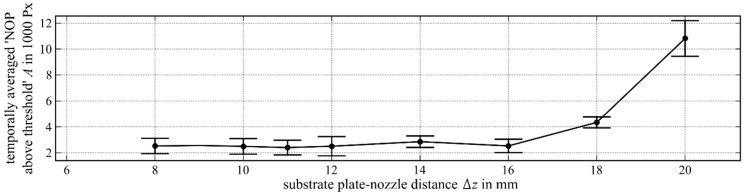
Temporally averaged NOP (intensity threshold 948 digits) over the variation of the substrate plate–nozzle distance.

Figure 9 shows the temporally averaged NOP over the variation of the substrate plate–nozzle distance. The temporally averaged NOP remained almost constant at about 2500 Px from the substrate plate–nozzle distance of Δz=8 mm up to Δz=16 mm. A a significant increase was recognisable from a distance of Δz>16 mm, so the temporally averaged NOP at a distance of Δz=20 mm was 10800 Px. The absolute temporal standard deviation followed a similar characteristic. It amounted to about 1220 Px at a substrate plate–nozzle distance of Δz<18 mm, but increased up to 2740 Px at a distance of Δz=20 mm.

## 4. Discussion

### 4.1. Oscillation of the Process at Lower Specific Energies

**Figure 10:**
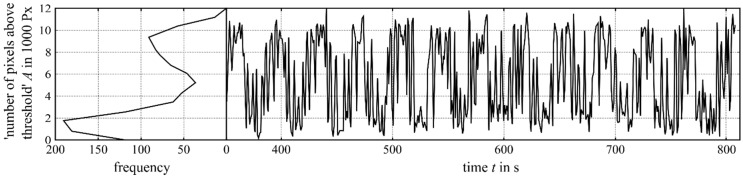
Extract of the NOP (intensity threshold 948 digits) during the variation of the laser power at a laser power of *P* = 1600 W. The histogram on the left side shows the whole steady state of the experiment.

**Figure 11:**
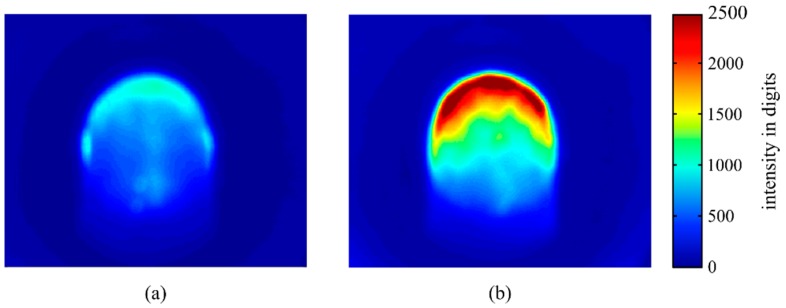
The average intensity distribution averaged over 10 frames in relation to (**a**) the lower NOP value range and (**b**) the upper value range recorded during the experiment at P=1400 W

By analysing the histograms of the steady-state time interval (Figure 10), we recognised a bimodal behaviour in the laser power values of P=1400 W and P=1600 W, which explains the high standard deviation in this range. The bimodality was caused by a change of the NOP between two value ranges. The time increment in which the NOP fluctuated around one mode lasted several seconds and depended on the laser power. At the laser power of P=1400 W, the NOP remained in the upper-value range (Figure 11a) for longer, while staying longer in the lower-value range (Figure 11b) at a power of P=1600 W. This behaviour was also recognisable through visual inspections of the welding process as an alternative of the weld pool brightness. 

The same bimodal behaviour was observed during the experiments in which the scanning speed was varied. It appeared at $v_{f}=1200\;\mathrm{mm}/\min$ and $v_{f}=1400\;\mathrm{mm}/\min$.

In order to compare the experiments of power variation with those of the scanning speed variation, the specific energy $E_{Spec}$ [13] was used to build an applicable characteristic value. The specific energy combined the absorbed laser power $P_{W}$ with the scanning speed $v_{f}$ and laser spot diameter d, according to $E_{Spec}=P_{W}/\left(v_{f}d\right)$.

To calculate the absorbed laser power $P_{W}$, the laser power had to be multiplied by the absorptance α (or emissivity ε) of the melt pool surface in accordance with $P_{W}=\alpha P$. Devesse et al. [7] showed that emissivity was approximately constant over the melt pool surface for the stainless steel LPW 316L. Furthermore, they determined the emissivity to lie in between 0.25 and 0.75. According to this, an absorptance of α=0.5 was assumed. 

Table 1 shows the experiments, during which the process was pulsating, and their respective specific energies. As one can see, the specific energy values during laser power variation correlated with those of the scanning speed variation. 

Steen et al. [13] determined a minimum specific energy of $E_{Spec}=22\;J/{\mathrm{mm}}^{2}$, which was required to achieve a stable process in the case of NiCr20Ti. At this point it can only be assumed that—due to a different heat transfer situation, the higher temperature of the base material and the different materials during the present analysis—lower specific energies were capable of achieving a stable process. This point should be examined in detail and might be a subject for further investigations.

### 4.2. Expression of the Plasma Plume

In addition, a further effect was observed regarding intensity distribution. An increasing laser power, as well as a decreasing scanning speed, resulted in a larger plasma plume. Again the specific energy is capable to compare the experimental series of varying the laser power and varying the scanning speed.

**Figure 12:**
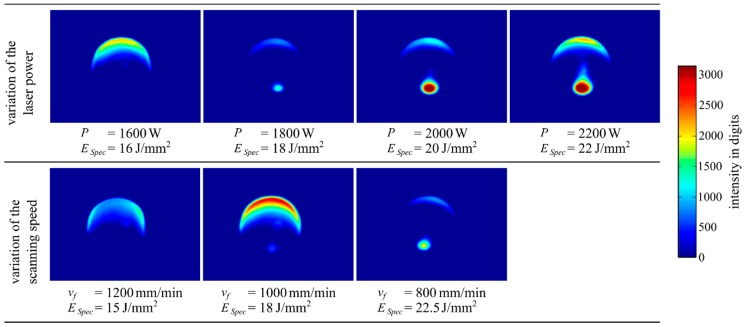
The averaged intensity distributions showing a growing expression of the plasma plume as the laser power increased and the scanning speed decreased.

Figure 12 shows no plasma plume at the specific energies $E_{Spec}<18\;J/{\mathrm{mm}}^{2}$. 

The expression of a plasma plume is a known phenomenon when the carrier and shielding gas argon is used in combination with higher specific energy values. For example, Ruiz et al. [11] varied the specific energy in the range of $E_{Spec}=15-30\;J/{\mathrm{mm}}^{2}$ while building up single tracks of NiCr19NbMo. They suspected the argon to form an ionised gas because of its first ionisation energy of $E_{Ion}=1520.8\;\mathrm{kJ}/\mathrm{mol}$.

### 4.3. Variation of the Powder Feed Rate

The introduced powder mass flow of $\dot{m}=8\;g/\min$ was insufficient to reach the required welding track height. This led to a growing distance between the base material and the nozzle tip. Therefore, the high temporally averaged NOP at this point was not an indicator of a low powder mass flow, but a result of the distance between the base material and nozzle tip being too large. Consequently, the powder mass flow affected the NOP indirectly, but the NOP only allowed limited conclusions about the powder mass flow to be drawn.

### 4.4. Variation of the Substrate Plate–Nozzle Distance

The behaviour of the NOPs over the variation of the substrate plate–nozzle distance can be explained by considering the self-curing effect. In order to prevent process errors according to a misestimated welding track height in the NC code, the powder focus was placed 2 mm below the laser focus (and the usual working distance). If the welding tracks were calculated too small, the process ran increasingly out of the powder focus and less powder reached the melt pool. If the welding tracks were calculated too high, the process drifted increasingly inside the powder focus, which led to higher powder efficacy and a higher welding track.

Figure 9 shows that the process was able to “cure” itself until a substrate plate–nozzle distance of Δz=16 mm. At higher distances, the self-curing effect was no longer able to compensate for it and the process drifted away. The high temporally averaged NOP at Δz=20 mm was caused by the fact that powder that could not reach the melt pool. In that case, power was ignited by the laser beam and burned brightly below the nozzle, emitting radiation that was not representative of the melt pool intensity.

### 4.5. Stochasticity of the Process

During the whole variation of the different process parameters, we recognise that the temporal standard deviation of the NOP was low at a stable process where the process parameter combination led to a well build-up, while it increased as the process became unstable. An exception can be seen at the high substrate plate–nozzle distance of Δz=20 mm, which is explained above.

## 5. Conclusions

In this paper a DMG MORI LT 65 3D *hybrid* machine, in combination with a camera-based coaxial temperature measurement system, was used to investigate which information of the intensity distribution of the melt pool surface were appropriate to draw conclusions about process conditions. The main results were as follows:

- For the material X2CrNiMo17-12-2 and the experimental configuration used, the minimum required specific energy could be determined at $E_{Spec}=18\;J/{\mathrm{mm}}^{2}$ during the steady state.
- At this point, the temporal standard deviation of the NOP (respectively the temporal variance) decreased and a light plasma plume could be detected qualitatively.
- A high NOP above an intensity threshold, together with its low relative temporal standard deviation, is a sign of an unstable cladding process. This is because of a working distance between the base material and the nozzle tip that is too large, and could serve as a release signal for an automatic machine switch-off.
- The analysis indicates that the NOP as a function of laser power needs to be in a certain region to ensure a stable process.
- The temporal standard deviation of the NOP gives additional information about process stability and can serve as an important characteristic measure.
- In general, it can be said that minimising process stochasticity for a given target NOP leads to stable process conditions.

## Figures and Tables

**Table 1:** Specific energy calculated for the process parameters, which led to a pulsating process.

**variation of the laser power**	P in W	1400	1600
	$v_{f}$ in mm/min	1000	1000
	$E_{Spec}$ in $J/{\mathrm{mm}}^{2}$	14.0	16.0
**variation of the scanning speed**	P in W	1800	1800
	$v_{f}$ in mm/min	1200	1400
	$E_{Spec}$ in $J/{\mathrm{mm}}^{2}$	15.0	12.9

## Appendix A

**Table A1:** Overview of the varied process parameters and the variation range.

Varied Parameter	Variation Range						
laser power P in W	1400	1600	1800	2000	2200	2400	
scanning speed $v_{f}$ in mm/min	800	1000	1200	1400	1600		
powder feed rate $\dot{m}$ in g/min	8	10	12	14	16	18	
substrate plate–nozzledistance Δz in mm	8	10	11	12	14	16	18
